# Large duodenal GIST with massive liver secondaries melting under Imatinib: a case report

**DOI:** 10.1186/1757-1626-1-197

**Published:** 2008-09-30

**Authors:** S Sankar, M Subramanian, T Arunkumar, N Venu, K Anand

**Affiliations:** 1 Department of Surgical gastroenterology, D-2 Private clinic, Sri Ramachandra Medical College & Research Center, Chennai, 600116, Tamilnadu, India; 2 Department of Surgical gastroenterology, D-2 Private clinic, Sri Ramachandra Medical College & Research Center, Chennai, 600116, Tamilnadu, India; 3 Department of Surgical gastroenterology, D-2 Private clinic, Sri Ramachandra Medical College & Research Center, Chennai, 600116, Tamilnadu, India; 4 Department of Surgical gastroenterology, D-2 Private clinic, Sri Ramachandra Medical College & Research Center, Chennai, 600116, Tamilnadu, India

## Abstract

Gastrointestinal stromal tumors(GIST) have become a well established entity and its taxonomy is no more ambiguous. Better understanding of the cell of origin and immunohistochmical markers have made this possible. Their treatment has been revolutionized with the advent of targeted molecular therapy, namely Imatinib mesylate. Herein we report a rare and interesting case of a thirty year old South Indian Lady with an extremely large Duodenal GIST with massive Liver secondaries. The phenomenon of metastatic GIST responding to Imatinib mesylate is not new. What is interesting in this case is the enormous tumor load at the time of presentation as exemplified by the cross sectional images. This kind of tumor response and patient survival deserves documentation

## Case presentation

**Figure 1:**
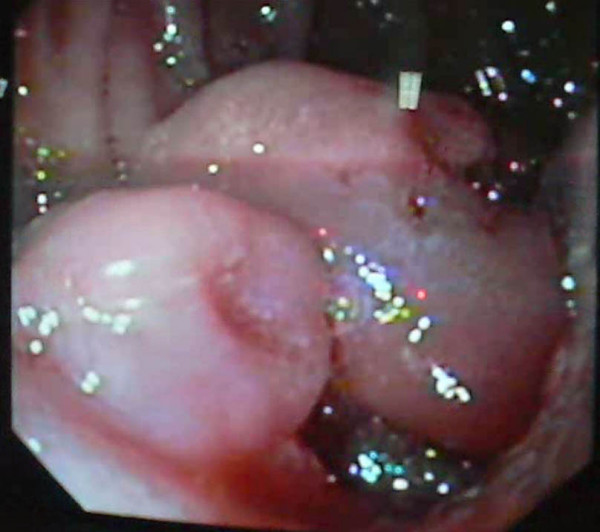
Endophytic fleshy tumor of duodenum.

**Figure 2:**
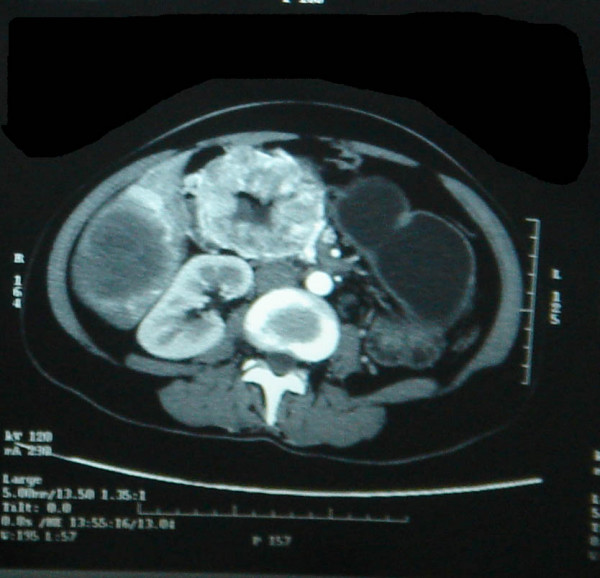
Large duodenal tumor with brisk contrast enhancement.

**Figure 3:**
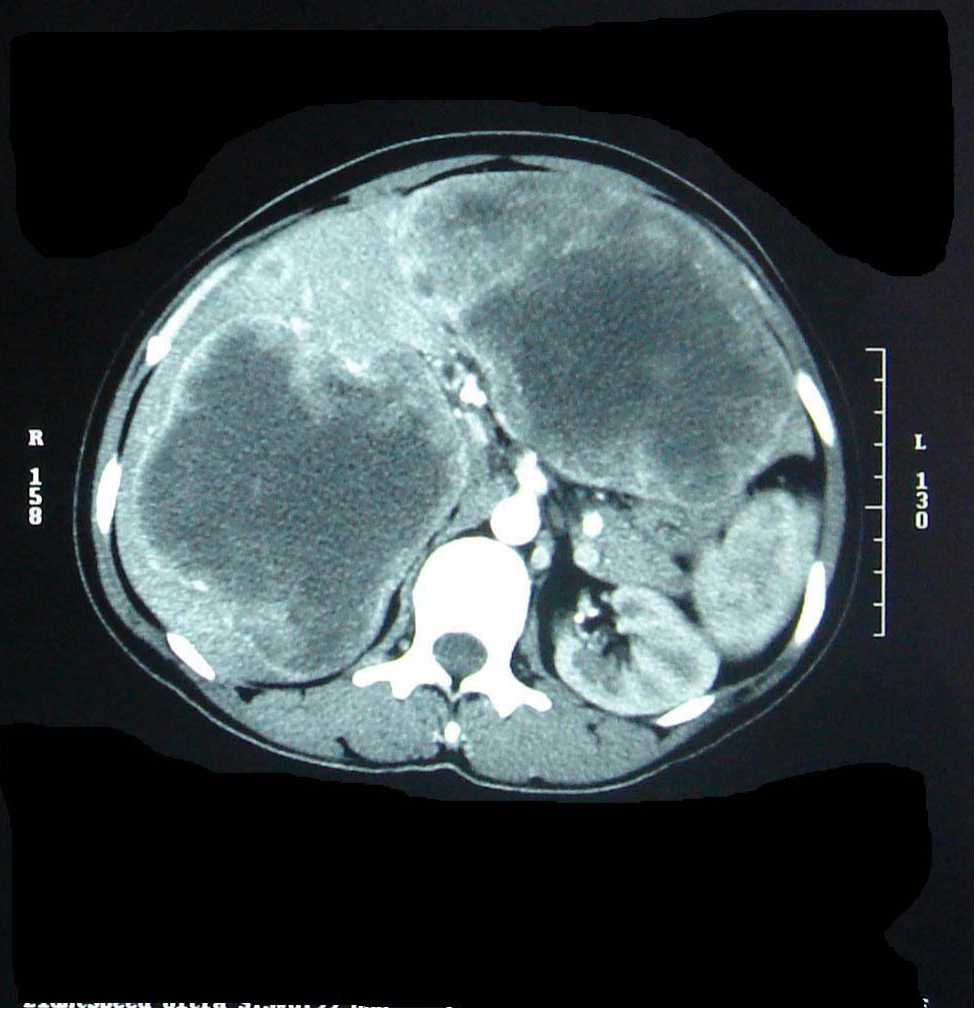
Massive bilobar liver metastasis.

Thirty year old South Indian Lady, a home maker presented to us in May 2005 with severe anemia and weight loss of three months duration. Her weight was 50 kg and height was 160 cm. She was neither an alcoholic nor a smoker. She had few episodes of malena during this period. She also had abdominal fullness, but no vomiting or pain. Clinical examination revealed a very pale patient with gross hepatomegaly with nodularity suggestive of secondaries Liver. Except for a grossly low hemoglobin, her other hemogram and biochemistry were within normal levels. Esophagogastroduodenoscopy picked up a fleshy protruding tumor in the second part of the duodenum(fig 1). CT abdomen showed a highly vascular and bulky tumor arising from the second part of the duodenum with massive secondary deposits in both the lobes of Liver(fig 2&3). Biopsies were done from the duodenal tumor and Liver metastasis. Histopathological report proved it to be Gastrointestinal stromal tumor(GIST) positive for CD 117.

**Figure 4:**
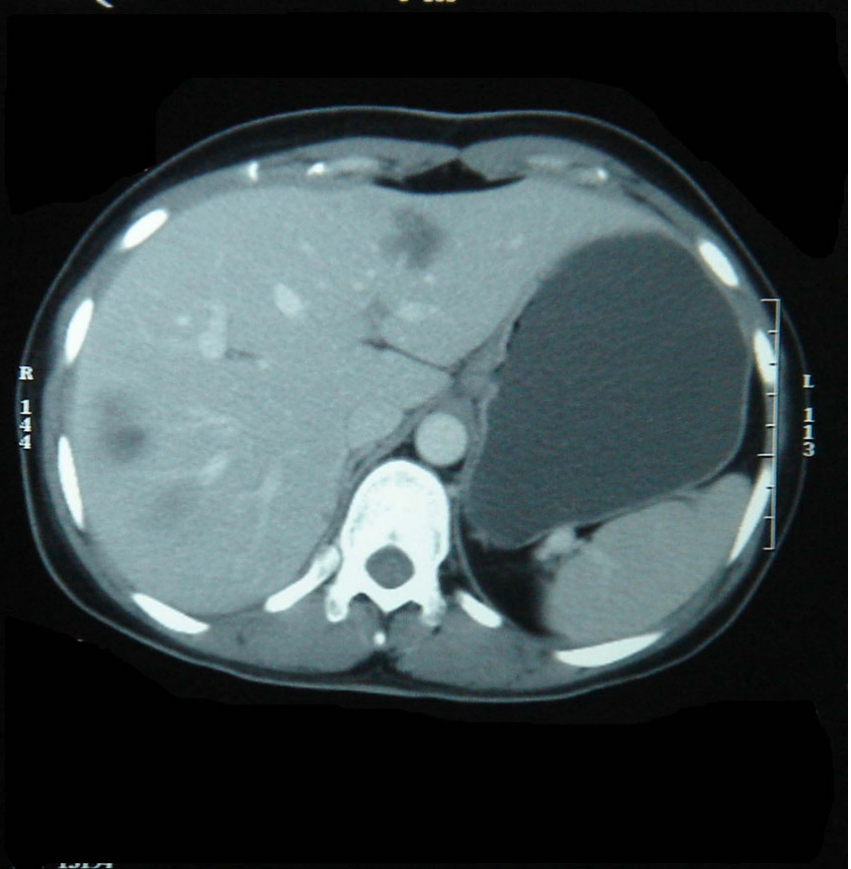
Dramatic response after Imatinib mesylate.

**Figure 5:**
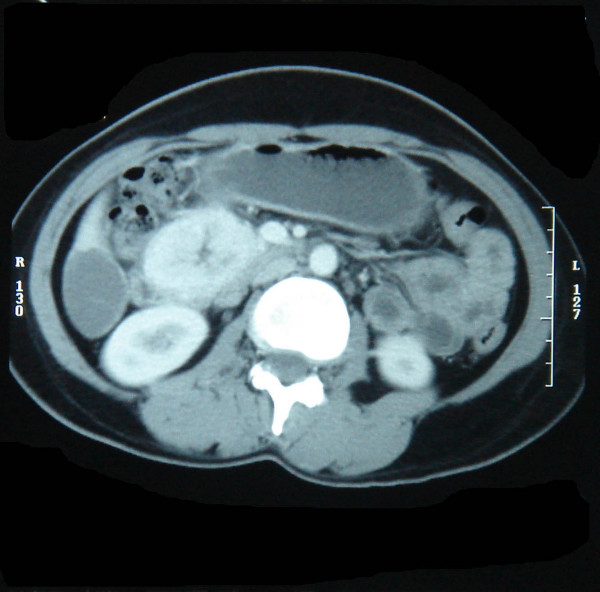
Partial response of duodenal tumor.

Anemia was corrected with blood transfusion. Since she had a metastatic GIST, she was started on Imatinib mesylate 400 mg once daily. The follow up CT showed a very dramatic response with almost complete clearance of metastatic deposit in the liver(fig 4). The duodenal tumor regressed in size(Fig 5). Since the Liver metastasis responded dramatically, we offered surgery for the primary duodenal tumor, which patient refused. At the point of writing this article, that is three years and six months after diagnosis, patient is fine and asymptomatic and continues to take Imatinib mesylate.

## Discussion

Gastrointestinal stromal tumor is not an uncommon entity in clinical practice with majority of them arising from stomach. Small intestine accounts for 20%–30% of the GIST. Majority of the small intestinal GIST arises from the jejunum and Ileum. Duodenum being the least common site. Duodenal GIST usually present with vague abdominal pain(50%–70%) or they bleed into the lumen(20%–50%)[1,2]. Small bowel GIST have a high propensity to exhibit malignant behavior[3]. Second part of the duodenum seems to be the common site of duodenal GIST and most of them will require pancreatoduodenectomy for complete resection[4]. Completely resected GIST has a five year survival of 30%–80%[5]. Incompletely resected tumors have a high recurrence rate(upto 90%). Historically Unresectable/metastatic GIST has a median survival of 12 months[6]. Imatinib mesylate, a specific tyrosikine kinase inhibitor has produced a paradigm shift in the treatment of GIST, due to the targeted molecular therapy. Imatinib produce sustained clinical response in more than 50% of the patients with advanced GIST and one year survival in these patients was 88%[7].

## Conclusion

Metastatic GIST responding to Imatinib is not an unusual phenomenon. But considering the sheer size and magnitude of the primary and secondary deposit, the response to imatinib has been immaculate. With the kind of tumor load this patient had, survival for more than three years is worth documenting. This case of GIST is rare and unusual for the following reasons

1. Duodenum being an uncommon site of GIST

2. Age of the patient 30 years. Usual age of GIST is beyond 40 years

3. Phenomenal cell kill achieved with imatinib

4. Despite the various poor prognostic factors patient continues to survive without any symptoms

## Competing interests

The authors declare that they have no competing interests.

## Authors' contributions

SS prepared the manuscript, KA edited the photos and all others read and approved the manuscript.

## Consent

Written and informed consent has been obtained from the patient for publication of the article and photos and the copy of the same is available for review by the editors.
